# Neonatal Pain-Related Stress Predicts Cortical Thickness at Age 7 Years in Children Born Very Preterm

**DOI:** 10.1371/journal.pone.0076702

**Published:** 2013-10-18

**Authors:** Manon Ranger, Cecil M. Y. Chau, Amanmeet Garg, Todd S. Woodward, Mirza Faisal Beg, Bruce Bjornson, Kenneth Poskitt, Kevin Fitzpatrick, Anne R. Synnes, Steven P. Miller, Ruth E. Grunau

**Affiliations:** 1 Pediatrics, University of British Columbia, Vancouver, British Columbia, Canada; 2 Developmental Neurosciences and Child Health, Child and Family Research Institute, Vancouver, British Columbia, Canada; 3 BC Mental Health and Addictions Research Institute, Vancouver, British Columbia, Canada; 4 Engineering Science, Simon Fraser University, Burnaby, British Columbia, Canada; 5 Psychiatry, University of British Columbia, Vancouver, British Columbia, Canada; 6 Radiology, University of British Columbia, Vancouver, British Columbia, Canada; 7 BC Children’s and Women’s Hospitals, Vancouver, British Columbia, Canada; 8 Pediatrics, Hospital for Sick Children and University of Toronto, Toronto, Ontario, Canada; Tokyo Metropolitan Institute of Medical Science, Japan

## Abstract

**Background:**

Altered brain development is evident in children born very preterm (24–32 weeks gestational age), including reduction in gray and white matter volumes, and thinner cortex, from infancy to adolescence compared to term-born peers. However, many questions remain regarding the etiology. Infants born very preterm are exposed to repeated procedural pain-related stress during a period of very rapid brain development. In this vulnerable population, we have previously found that neonatal pain-related stress is associated with atypical brain development from birth to term-equivalent age. Our present aim was to evaluate whether neonatal pain-related stress (adjusted for clinical confounders of prematurity) is associated with altered cortical thickness in very preterm children at school age.

**Methods:**

42 right-handed children born very preterm (24–32 weeks gestational age) followed longitudinally from birth underwent 3-D T1 MRI neuroimaging at mean age 7.9 yrs. Children with severe brain injury and major motor/sensory/cognitive impairment were excluded. Regional cortical thickness was calculated using custom developed software utilizing FreeSurfer segmentation data. The association between neonatal pain-related stress (defined as the number of skin-breaking procedures) accounting for clinical confounders (gestational age, illness severity, infection, mechanical ventilation, surgeries, and morphine exposure), was examined in relation to cortical thickness using constrained principal component analysis followed by generalized linear modeling.

**Results:**

After correcting for multiple comparisons and adjusting for neonatal clinical factors, greater neonatal pain-related stress was associated with significantly thinner cortex in 21/66 cerebral regions (*p*-values ranged from 0.00001 to 0.014), predominately in the frontal and parietal lobes.

**Conclusions:**

In very preterm children without major sensory, motor or cognitive impairments, neonatal pain-related stress appears to be associated with thinner cortex in multiple regions at school age, independent of other neonatal risk factors.

## Introduction

Altered brain development is evident in children born very preterm (24–32 weeks gestational age) in early infancy [1]–[3] and at school-age [4], [5], including reduced gray and white matter volumes in infancy, childhood and adolescence [6]–[10], compared to term-born peers. Normal brain growth involves changes in cortical thickness reflecting cellular maturational changes related to myelination and synaptic pruning [11]. Recent findings from our group, in a separate cohort, showed delayed microstructural development of the cortical gray matter in very preterm neonates at term equivalent [12]. Children born preterm show altered cortical thickness in childhood and adolescence [7], [13]–[18]. Specifically, thinner cortex has been reported in superior and temporal, middle frontal, anterior cingulate cortex, supramaginal, precuneus, and post central regions when compared to term born controls at school age [7], [14]. In adolescents born preterm, thinner cortex has been reported in the enthorhinal, temporal, and parietal regions [13], [16], [18]. Thicker cortex has been reported in preterm children with periventricular leukomalacia, identified on neonatal ultrasound and still evident on magnetic resonance (MR) images at school age [14]. Little is known about the etiology and possible clinical risk factors underlying regional cortical thinning in children born preterm who do not have major brain injury.

Early-life adversity in animal models, including maternal separation [19]–[22] and pain exposure [23]–[27], induces long-term changes in brain and behavior (reviews [28], [29]). Infants born very preterm are exposed to weeks to months of hospitalization in the neonatal intensive care unit (NICU) during a period of rapid neuronal proliferation and cell differentiation (e.g. differentiation of subplate neurons, formation of synapses, selective pruning of neuronal processes and synapses). Repeated procedures inducing pain-related stress place very preterm infants at particular risk due to the very rapid brain development and programming of stress systems while they are in the NICU [30], [31]. Given our previous findings that pain-related stress was associated with atypical brain development in the neonatal period [8], our aim was to evaluate whether neonatal pain-related stress, quantified as the number of skin-breaking procedures adjusted for confounding clinical factors, is associated with variations in cortical thickness at age 7 years in a different cohort of children born very preterm.

We hypothesized that greater neonatal pain-related stress would be related to reduced cortical thickness, after adjusting for confounding neonatal clinical factors associated with premature birth and neonatal care such as gestational age, number of days on mechanical ventilation, early severity of illness, surgery, infection, and morphine exposure.

## Materials and Methods

### 1. Study Design and Participants

Participants were part of a larger longitudinal study of long-term effects of neonatal pain-related stress on neurodevelopment of children born very preterm (24–32 weeks gestation) e.g. [31], [32], who were admitted to the level III NICU at British Columbia’s Women’s Hospital between 2000 and 2004. Out of 106 children seen at age 7 years, 43 refused magnetic resonance imaging (MRI), leaving 63 school-age children, 2 were not scanned, 61 underwent MRI. From the 61 preterm children that underwent MRI scans at age 7 years, 13 were excluded due to poor quality MR images due to movement artifact and one for missing neonatal clinical data. In addition, two children with periventricular leukomalacia (PVL) and/or intraventricular hemorrhage (IVH) grade 3 or 4 on neonatal ultrasound and confirmed on MR at age 7 years (author KP) were excluded, and three left handed children were excluded to eliminate the effect of handedness on cortical thickness asymmetries [33], [34]. We included in our study three very preterm infants diagnosed on neonatal ultrasound with IVH grade 1 or 2 (two infants with IVH grade 1) and six children that showed minimal to moderate white matter injury (≤3 lesions) on MR scans at school-age, one of which had IVH grade 1 on neonatal ultrasound. None of the cortical thickness measures for these children were outliers compared to the rest of the sample, therefore they were included in the study. All of the children scanned had an IQ score above 70 on the Wechsler Intelligence Scale for Children –4^th^ Ed [35], and none had a major sensory or motor impairment. The final study sample comprised 42 right-handed children (38% boys).

The study was approved by the Clinical Research Ethics Board of the University of British Columbia and the British Columbia Children’s and Women’s Research Ethics Board. Written informed consent was obtained from parents and assent from children.

### 2. Procedures

#### Magnetic resonance imaging

MRI was performed using a standard 12 channel head coil on a Siemens 1.5 Tesla Avanto (Berlin, Germany) with VB 16 software. The following images were acquired: a 3D T1 weighted SPGR sequence 18/9.2/256/1 mm/0/256×256 (TR/TE/FOV/Thickness/Gap/Matrix), axial FSE T2 4030/90/220/3 mm/0.1 mm/512×354, axial FLAIR 8900/87/5 mm/1 mm/256×154 and a 12 direction DTI sequence 7800/82/256/2 mm/0/128×128 using B values of 700 and 1000. All imaging sessions were performed without sedation. On the study day, each child first had a session in a mock scanner to acclimatize to the noise and feeling of being in a MRI scanner, followed by the actual study scan. Children were instructed to remain still and watched a video during the sessions that lasted approximately 30 minutes.

An experienced pediatric neuroradiologist (KP), blinded to the child’s medical history, assessed the MR scans for ventriculomegaly, cerebellar hemorrhage and severity of white matter injury, as previously described [36]. No child had a severe brain injury at school age (i.e. no cerebellar hemorrhage, ventriculomegaly or severe white matter injury [i.e. >3 lesions or 2 with 5% hemisphere involved]).

#### Cortical thickness measures

Regional cortical thickness was calculated using a topology aware technique originally proposed by Gibson *et al.* [37] which modifies the normally accepted method of calculating the thickness as the length of the streamlines obtained from the well-established and validated Laplacian streamlines method [38], [39]. This implementation uses FreeSurfer surfaces to enforce separation of volumetric domains, which is important where adjacent gyri/sulci may abut. In the original implementation, the abutting gyri/sulci can lead to overestimation of cortical thickness, which is avoided in this custom implementation, thereby giving more accurate thickness in regions where topological issues such as touching gyri occur, and no difference in thickness calculated in other regions. For detecting group-wise maps of statistically significant thickness changes, FreeSurfer and Laplacian-streamlines based volumetric methods lead to similar results [40]. The coupled surface method establishes the inner surface initially and propagates that mesh to another surface thus preserving the corresponding points incorporating the topology dictated by the inner and the outer surfaces of the gray matter in the volumetric Laplacian streamline thickness computation as described in [41] as the arc length of the streamlines of the solution of Laplace’s equation on the cortical mantle.

Cortical thickness was calculated for each individual brain using the segmentation output from the preprocessing step, where the output obtained is the pial surface for each subject where each vertex has a thickness value associated with it. The surfaces used to define the outer and inner boundaries in our methods were constructed using an automated cortical surface reconstruction method developed by Dale and Fischl [42], [43]. The 66 brain regions of interest (ROIs) used in this study were parcellated by FreeSurfer using the Desikan-Killiany Atlas, an automated labeling system developed and validated by Desikan *et al*. [44].

#### Clinical data collection

Medical and nursing chart review of neonatal data from birth to term equivalent was carried out by highly trained neonatal research nurses. Data collected included, but was not limited to, birth weight, gestational age (GA), number of days on mechanical ventilation and/or oscillation, illness severity on day 1 (Score for Neonatal Acute Physiology [SNAP]- II [45]), number of surgeries, presence of culture proven infection, and cumulative dose of morphine. The cumulative dose of morphine was calculated (intravenous dose plus converted oral dose) as the average daily dose adjusted for daily body weight, multiplied by the number of days the drug was given, as we have used previously [8], [31]. We quantified neonatal pain-related stress as the number of skin-breaking procedures (e.g., heel lance, peripheral intravenous or central line insertion, chest-tube insertion, tape removal, and nasogastric tube insertion) during the stay in the NICU, as previously used [8], [31], [46]. Each attempt at a procedure was counted as one skin-break; all nursing staff in our NICU have been trained to precisely record each attempt.

### 3. Statistical Analysis

Principal component analysis (PCA) is a method of data reduction of large sets of related variables by reducing them to a few vectors of weightings that best explain the variance, while losing as little information as possible. Each extracted vector, referred to as a “component”, accounts for a portion of the total variance in the data: the first component accounts for the largest amount of variance, with each successive component accounting for a smaller amount of the total variance. Constrained principal component analysis (CPCA) combines multivariate multiple regression and PCA, and allows examination of the component structure of the variance in a set of dependent variables that is specifically predicted by a set of predictor variables [47]–[49]. CPCA is two-step process, referred to as the external and internal analysis. The external analysis consists of a multivariate least squares multiple regression of the dependent measures on the independent measures, producing predicted and residual scores for each dependent measure. In the present study, the matrix of predicted scores reflects the variance in cortical thickness that can be accounted for by the neonatal clinical variables, and the residual matrix reflects the variance that cannot be accounted for by the neonatal clinical variables. Seven neonatal clinical predictors were included: number of skin-breaking procedures, gestational age, number of days on mechanical ventilation, illness severity on day 1 (SNAP-II), number of surgeries, culture proven infection, and cumulative morphine exposure adjusted for daily weight. The internal analysis consists of PCAs on each of the aforementioned matrices. The resulting component solutions (overall, predicted, and residual solutions) are examined to determine which dimensions of the cortical thickness can be explained by the neonatal clinical variables. To determine the particular neonatal clinical predictors that are related to each of the components extracted from the predicted solution, correlations were computed between each component score and the set of independent variables (i.e., neonatal clinical data). The number of components retained for each PCA was determined by inspection of scree plots. All PCA solutions were separately rotated using varimax with Kaiser normalization. Solutions were bootstrapped 1000 times by Monte Carlo methods to produce confidence intervals and *p*-values [50], [51]. Computations for CPCA and bootstrapping were carried out using MATLAB version 7.6 (The MathWorks, 2008, Natick, MA).

Then, to further examine the individual contribution of each neonatal predictor on the measured variations of cortical thickness, we conducted a generalized linear (GENLIN) modeling analysis, adjusting for neonatal clinical factors (GA, SNAP-II at day 1, culture proven infection, number of days on mechanical ventilation, number of surgeries, cumulative morphine exposure adjusted for daily weight, and number of skin-breaking procedures). GENLIN provides an extension of general linear models and relaxes the requirement of equality or constancy of variances that is required in traditional linear models. GENLIN was carried out using the Statistical Package for Social Sciences (SPSS) version 16.0 (IBM, Somers, NY). False discovery rate (FDR 5%) [52] was employed to correct for multiple comparisons between brain regions for CPCA and GENLIN.

## Results

Demographic and neonatal clinical data are presented in Table 1. Children were scanned at a median age of 7.8 years (interquartile range [IQR] 7.7–8). During their NICU stay, these children underwent a median of 74 (IQR 45–136) skin-breaking procedures.

**Table 1:** Demographic and neonatal characteristics.

Characteristics	n = 42 (16 boys,26 girls)
**Neonatal characteristics**	
GA at birth (weeks)	29.71 (27.29–31.57)
Birth Weight (grams)	1203 (877–1558)
SGA (number, %)	5 (12%)
IVH grade I–II (number, %)	3 (7%)
Illness severity day 1 (SNAP-II)	8.5 (0–14.75)
Skin-breaking procedure (number)	74 (45–136)
Mechanically ventilated (number, %)	25 (59.5%)
Days of mechanical ventilation (number)	2 (0–10)
Culture proven infection (number, %)	11 (26)
Surgery ≥1 (number, %)	8 (19)
Morphine (dose in µg^1^)	0.00 (0–664)
Non-mechanically ventilated	0.00 (0–0)
Mechanically ventilated	205 (42–1332)
**School Age characteristics at scan**	
Chronological Age (years)	7.76 (7.71–8.04)
Weight (kg)	23.1 (21.3–26.3)
Height (cm)	124 (120–126.6)
Head circumference (cm)	51.5 (50–52.7)
Mild/moderate white matter injury(number, %)^2^	6 (14%)
WISC-IV Full scale score (IQ)	101 (91–108)

Median and interquartile range are given unless otherwise specified.

1 Cumulative daily dose adjusted for daily body weight.

2 Mild to moderate white matter injury (≤3 lesions) on MR scan at school age (1 child with mild white matter injury at 7 years had IVH grade 1 on neonatal ultrasound).

GA, gestational age; SGA, small for gestational age (<10%tile); %, percent; IVH, intraventricular hemorrhage grade I–II diagnosed on neonatal ultrasound; SNAP-II, score for neonatal acute physiology; WISC-IV, Wechsler Intelligence Scale for Children –4^th^ Ed.

Since child age may contribute to variations in cortical development [11], we performed correlations between age at MR scan and cortical thickness and no statistically significant associations were found, after Bonferroni correction for multiple comparisons. Given that gender differences in cortical thickness have been reported in adolescence [53], we conducted independent t-tests comparing cortical thickness in boys and girls, finding no statistically significant differences in any of the 66 brain regions assessed, after Bonferroni correction for multiple comparisons. It was noted that without correction, cortical thickness differed by gender only in 1/66 regions.

### 1. Constrained Principal Component Analysis (CPCA)

Given the limited number of participants, the high number of brain regions examined, and correlated neonatal clinical factors, we first examined the relationships between the neonatal clinical variables and cortical thickness in all brain regions. This permitted unbiased handling and interpretation of our data.

Table 2 shows the distribution of variance of the overall, predicted and residual solutions of the CPCA. Three components were extracted from the predicted solutions (eigenvalues 14.77, 2.46, and 1.56 respectively), which corresponds to 13.4% of the overall variance for component 1, 7.5% and 6.9% for components 2 and 3 respectively. The external analysis showed that the 7 clinical predictors accounted for 32.9% of the overall variance in cortical thickness.

**Table 2:** Constrained principal component analysis results to explain variance in cortical thickness in relation to neonatal clinical factors.

	External Analysis (Regression)	Internal Analysis (PCA)			
Variance	Total	Comp 1	Comp 2	Comp3	1+2+3
Overall	67.61	13.70	12.34	11.60	37.64
*% Overall*	*100%*	*20.26%*	*18.26%*	*17.16%*	*55.68%*
Predictable	22.21	9.04	5.10	4.66	18.79
*% Predictable*	*100%*	*40.68%*	*22.95%*	*20.96%*	*84.59%*
*% Overall*	***32.85%***	***13.36%***	***7.54%***	***6.89%***	***27.79%***
Residual	45.40	10.62	9.13		19.75
*% Residual*	*100%*	*23.39%*	*20.10%*		*43.50%*
*% Overall*	*67.15%*	*15.71%*	*13.50%*		*29.21%*

Comp = Component; PCA = principal component analyses; eigenvalues for 3 components = 14.77, 2.46, 1.56 respectively.

In Principal component analysis (PCA), a component refers to a vector of weightings that best explain the variance. Each extracted component accounts for a portion of the total variance in the data: the first component accounts for the largest amount of variance, with each successive component accounting for a smaller amount of the total variance.

In Constrained Principal Component Analysis, the external analysis consisted of a multivariate multiple regression of the predictor variables on the dependent measures, which produces predicted and residual scores. In the present study, the matrix of predicted scores reflects the variance in cortical thickness that is predicted from the 7 neonatal clinical variables, and the residual matrix reflects the variance that is not predicted by these variables.

The internal analysis consisted of three different PCAs: one on the unconstrained variance in cortical thickness (Overall), one on the variance in the cortical thickness predictable from the 7 neonatal clinical variables (Predictable), and one on the variance in cortical thickness not predictable from the clinical variables (Residual). The variance accounted for by the external analysis and each component extracted in the internal analysis is listed in regular font. The percentages of variance accounted for by the external analysis and each component extracted in the internal analysis are listed in *italic* font. All internal analyses were separately rotated using varimax.

Component loadings and their bootstrapped confidence intervals and *p*-values in each brain regions of interest (ROIs) are summarized in Table 3. Both the first and second components were characterized by negative loadings on all brain regions (i.e. decreased cortical thickness), while the third component had positive loadings (i.e. increased cortical thickness). The dominant loadings on the first component were distributed prominently on various frontal and central brain regions. On the second component, left supramarginal, bilateral inferior parietal, and right lateral occipital ROIs had the largest loadings. Bilateral caudal anterior cingulate and left lingual ROIs had the largest loadings for the third component.

**Table 3:** Brain regions with loadings for the 3 components.

Component	Brain Regions	Componentloadings	*p-*value	lower CI	upper CI
1	left precentral	−0.65	<0.0001	0.39	0.89
1	left superior frontal	−0.62	<0.0001	0.35	0.87
1	right superior frontal	−0.54	<0.0001	0.28	0.78
1	left superior parietal	−0.60	<0.0001	0.31	0.88
1	left inferior parietal	−0.56	<0.0001	0.28	0.82
1	left post central	−0.50	<0.0001	0.25	0.74
1	right superior parietal	−0.52	<0.0001	0.26	0.78
1	left supramarginal	−0.44	0.0001	0.21	0.66
1	right inferior parietal	−0.52	0.0002	0.24	0.79
1	left frontal pole	−0.54	0.0003	0.25	0.83
1	left precuneus	−0.48	0.0003	0.22	0.73
1	right rostral middle frontal	−0.49	0.0005	0.21	0.75
1	left rostral middle frontal	−0.47	0.0005	0.20	0.73
1	right pars orbitalis	−0.49	0.0005	0.21	0.76
1	right middle temporal	−0.51	0.0007	0.21	0.79
1	left caudal middle frontal	−0.50	0.0008	0.21	0.79
1	right caudal middle frontal	−0.57	0.0009	0.23	0.90
1	right supramarginal	−0.48	0.001	0.19	0.75
1	right precuneus	−0.44	0.002	0.16	0.71
1	right inferior temporal	−0.43	0.002	0.16	0.69
1	right pars opercularis	−0.44	0.003	0.15	0.72
1	right superior temporal	−0.48	0.003	0.16	0.78
1	left paracentral	−0.57	0.003	0.20	0.94
1	right paracentral	−0.54	0.004	0.17	0.90
1	right precentral	−0.55	0.006	0.16	0.94
1	left pars triangularis	−0.38	0.006	0.11	0.65
1	left middle temporal	−0.38	0.009	0.09	0.65
1	right postcentral	−0.44	0.01	0.10	0.76
1	right lateral orbitofrontal	−0.37	0.01	0.09	0.65
1	left pars opercularis	−0.33	0.01	0.07	0.58
1	right pars triangularis	−0.41	0.01	0.09	0.73
1	left entorhinal	−0.37	0.02	0.06	0.66
2	left inferior parietal	−0.48	0.0003	0.22	0.74
2	left inferior temporal	−0.46	0.0008	0.19	0.72
2	right inferior parietal	−0.51	0.0008	0.21	0.80
2	right lateral occipital	−0.54	0.001	0.21	0.85
2	left supramarginal	−0.55	0.003	0.19	0.90
2	left precuneus	−0.31	0.003	0.10	0.51
2	left medial orbitofrontal	−0.44	0.004	0.14	0.73
2	left superior parietal	−0.38	0.005	0.11	0.63
2	right superior parietal	−0.39	0.006	0.11	0.65
2	right inferior temporal	−0.36	0.006	0.10	0.61
3	left caudal anterior cingulate	0.56	0.0001	−0.84	−0.28
3	left lingual	0.54	0.0005	−0.83	−0.23
3	right pars opercularis	0.42	0.002	−0.67	−0.15
3	right caudal anterior cingulate	0.57	0.003	−0.93	−0.19
3	left posterior cingulate	0.38	0.004	−0.63	−0.12
3	left rostral anterior cingulate	0.46	0.004	−0.76	−0.15
3	right rostral middle frontal	0.38	0.004	−0.64	−0.12
3	left lateral orbitofrontal	0.33	0.005	−0.55	−0.10
3	right pars triangularis	0.32	0.005	−0.54	−0.09
3	left fusiform	0.37	0.007	−0.64	−0.10
3	left inferior temporal	0.30	0.01	−0.52	−0.07

C.I = 95% confidence interval; only *p*-values with less than 5% false discovery rate are listed.

The variance in cortical thickness explained by the 7 neonatal clinical factors within each component is shown in Table 4. The external analysis in CPCA showed that pain-related stress was the strongest predictor from our set of 7 neonatal factors. The number of skin-breaking procedure loaded highly on the first two components, infection loaded significantly only on component 1, while surgery significantly loaded uniquely and predominately on component 2. Morphine exposure and duration of mechanical ventilation significantly loaded on all three components but dominated the 3^rd^ component. Severity of illness on day 1 (SNAP-II) loaded significantly on all 3 components. Component 1 suggests that the number of skin-breaking procedures during NICU care, illness severity on day 1, culture-proven infection, gestational age, cumulative morphine exposure, and days on mechanical ventilation are related to reduced cortical thickness, primarily in frontal and parietal regions. Component 2 in addition, captured predominately surgery, but not infection, and is related to reduced cortical thickness, primarily in parietal, temporal and occipital regions. Component 3, contrary to the two other components, was positively associated with cortical thickness and primarily reflected cumulative morphine exposure and number of days on mechanical ventilation. Therefore, we interpreted component 1 as procedural and inflammation related pain, component 2 as surgery related pain, and component 3 as primarily morphine exposure. Since both components 1 and 2 had negative loadings on all brain regions, this implied that higher procedural/inflammatory and surgical pain were associated with a reduction in cortical thickness predominately in the frontal and parietal regions for component 1, and parietal, temporal and occipital regions for component 2. In contrast, component 3 showed a positive association with cortical thickness, predominately within the anterior cingulate cortex ROIs, suggesting thicker cortex in association with higher cumulative morphine exposure in these regions.

**Table 4:** Constrained principal component analysis loadings for the 7 neonatal clinical factors to explain variance in cortical thickness.

	Component 1			Component 2			Component 3		
	Loading	*p*-value	C.I	Loading	*p*-value	C.I	Loading	*p*-value	C.I
Pain	**0.67**	<0.0001†	[0.37, 0.98]	**0.57**	0.0003†	[0.26, 0.87]	−0.06	0.70	[−0.37, 0.25]
Morphine	**0.38**	0.02†	[0.08, 0.68]	**0.62**	<0.0001†	[0.32, 0.93]	**0.59**	<0.0001†	[0.29, 0.88]
Surgery	0.05	0.75	[−0.26, 0.36]	**0.92**	<0.0001†	[0.62, 1.23]	0.28	0.07	[−0.02, 0.58]
Ventilation	**0.32**	0.04†	[0.02, 0.63]	**0.63**	<0.0001†	[0.32, 0.94]	**0.57**	0.0002†	[0.26, 0.87]
SNAP-II	**0.75**	<0.0001†	[0.46, 1.06]	**0.32**	0.04†	[0.01, 0.63]	**0.36**	0.02†	[0.05, 0.67]
Infection	**0.47**	0.002†	[0.17, 0.78]	0.04	0.79	[−0.26, 0.34]	0.001	0.99	[−0.31, 0.31]
GA	−**0.48**	0.002†	[−0.79, −0.18]	−**0.64**	<0.0001†	[−0.94, −0.34]	0.02	0.92	[−0.28, 0.31]

Predictor loadings were computed as correlations between component scores and the set of neonatal clinical variables.

C.I. = 95% confidence interval; Pain = number of skin-breaking procedures exposure; Morphine = cumulative daily dose in milligrams adjusted for daily body weight; Ventilation = number of days on mechanical ventilation; Surgery = number of surgeries; Infection = number of culture proven infection; SNAP-II = score for neonatal acute physiology; GA = gestational age.

† *p*-value threshold for significance adjusted for multiple comparisons with a false discovery rate (FDR) correction set at 5%; *p*-values and confidence intervals were computed by bootstrapping 1000 times.

To check whether birth weight (BW) might provide important additional information beyond GA in explaining variations in cortical thickness, we added BW to the set of neonatal factors in a further CPCA analysis. With the inclusion of BW and GA, the overall predictable variance accounted for by the neonatal factors increased by only 1.8%. Given the low sample size and the high correlation between GA and BW (*r* = 0.76, *p*<0.0001) we only retained GA in further analyses.

### 2. Generalized Linear Modeling (GENLIN)

**Figure 1:**
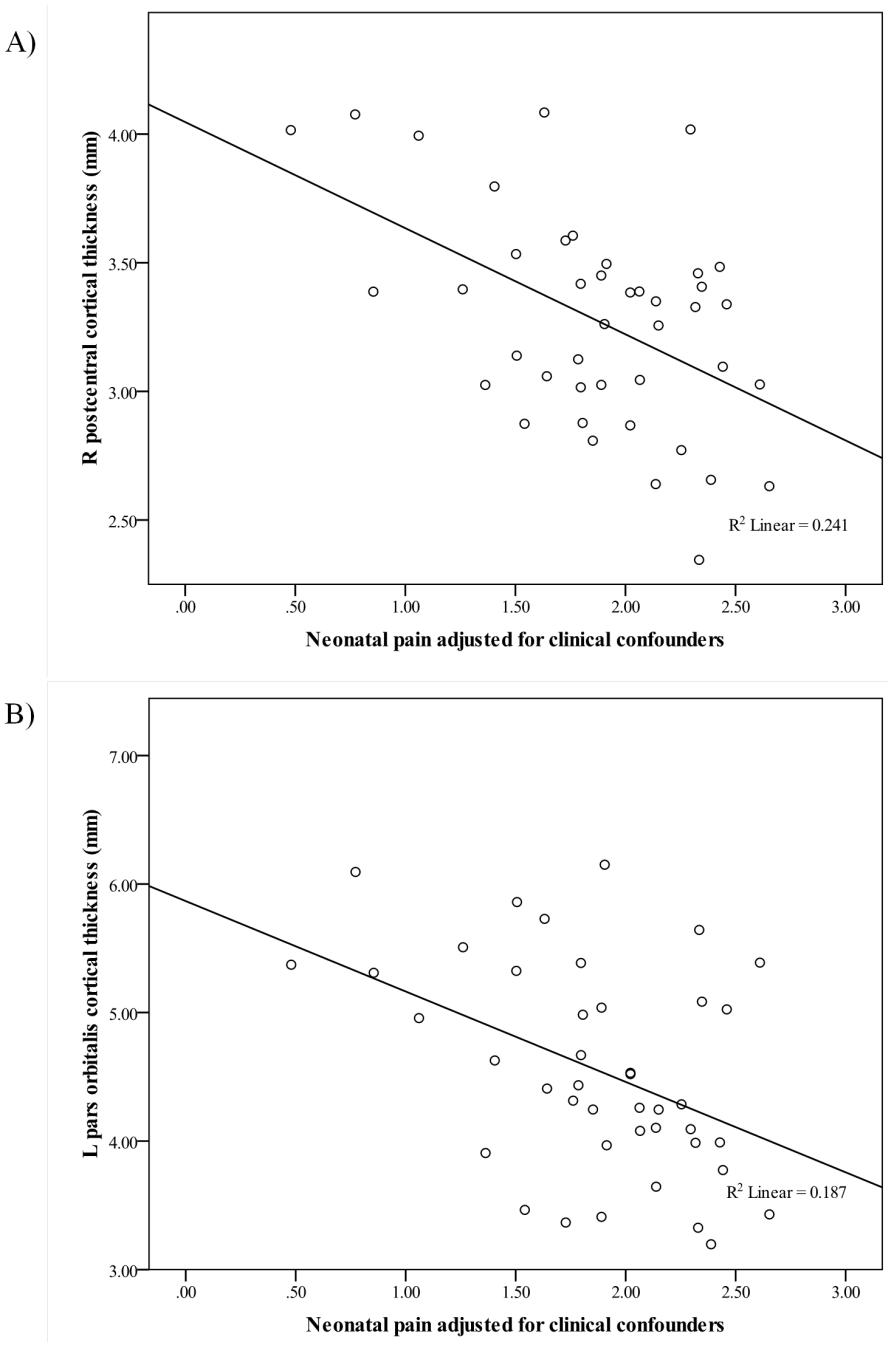
Cortical thickness at age 7 years in relation to neonatal pain-related stress adjusted for clinical confounders. Cortical thickness in two of the brain regions where most significant (*p*≤0.0001) thinning in relation to neonatal pain-related stress was found. A) Scatter plot of right hemisphere postcentral cortical thickness in relation to neonatal pain-related stress (number of skin-breaking procedures log transformed) adjusted for gestational age, severity of illness on day 1, number of culture proven infection, number of days on mechanical ventilation, number of surgeries, and cumulative daily morphine dose in milligrams adjusted for daily body weight. B) Scatter plot of left hemisphere pars orbitalis cortical thickness in relation to neonatal pain-related stress (same as in A).

Neonatal clinical factors were inspected for normality, then when necessary were log transformed (neonatal skin-breaking procedures and number of days on mechanical ventilation). Using the findings from the CPCA, a GENLIN model for each of the 66 brain regions was conducted, corrected for multiple comparisons (i.e. 5% FDR). After adjusting for neonatal clinical factors (i.e. gestational age, SNAP-II at day 1, infection, number of days on mechanical ventilation, number of surgery, cumulative morphine exposure), greater number of neonatal skin-breaking procedures was significantly associated with reduced cortical thickness in 21 out of the 66 brain regions assessed (*p*-values ranged from 0.00001 to 0.014; Table 5), *p*-value threshold for significance adjusted for multiple comparisons with a FDR correction set at 5%. Most significant (*p*≤0.001) thinning in relation to neonatal pain-related stress was found in bilateral postcentral, superior frontal, rostral middle frontal, left hemisphere precentral and pars orbitalis, as well as the right hemisphere supramarginal region. Of all the neonatal clinical factors, pain-related stress (adjusted for neonatal clinical factors) was the most robust independent predictor of regional variation in cortical thickness. The association between neonatal pain-related stress (adjusted for confounders) and right hemisphere postcentral and left pars orbitalis cortical thickness are shown as examples (Figure 1).

**Table 5:** GENLIN results for the 21/66 cortical regions significantly associated with neonatal pain-related stress (adjusted for clinical confounders).

	Infection		Pain		Ventilation		Morphine		SNAP-II		Surgery		GA	
Brain Regions	B	*p*-value	B	*p*-value	B	*p*-value	B	*p*-value	B	*p*-value	B	*p-*value	B	*p*-value
L entorhinal	−0.34	0.15	−1.13	**0.003** †	0.48	0.11	−0.06	0.90	−0.01	0.13	−0.19	0.29	−0.01	0.86
L pars orbitalis	0.16	0.34	−1.03	**<0.0001** †	0.62	0.004	0.33	0.30	−0.004	0.57	−0.06	0.64	0.04	0.32
R pars orbitalis	−0.24	0.27	−0.85	**0.014** †	0.18	0.50	1.08	0.01	−0.03	**0.001** †	−0.15	0.35	0.03	0.50
R rostral middle frontal	−0.06	0.63	−0.65	**0.001** †	0.18	0.25	0.67	0.01	−0.01	0.01	−0.15	0.11	0.01	0.70
R superior frontal	−0.06	0.53	−0.63	**<0.0001** †	0.19	0.14	0.24	0.22	−0.01	0.02	−0.06	0.48	0.002	0.92
R supramarginal	−0.03	0.68	−0.58	**<0.0001** †	0.24	0.01	0.35	0.02	−0.004	0.22	−0.07	0.22	0.04	0.06
L superior frontal	−0.02	0.84	−0.56	**0.001** †	0.17	0.18	0.25	0.20	−0.01	0.02	0.02	0.79	0.02	0.40
R postcentral	0.06	0.48	−0.55	**<0.0001** †	0.20	0.07	0.15	0.36	−0.002	0.55	−0.04	0.55	−0.003	0.89
L precentral	−0.06	0.51	−0.54	**<0.0001** †	0.16	0.17	0.09	0.62	−0.01	0.004	−0.002	0.98	−0.01	0.76
R inferior temporal	0.04	0.83	−0.54	**0.006** †	0.21	0.17	0.30	0.18	−0.01	0.16	−0.13	0.17	0.02	0.49
L lingual	0.01	0.90	−0.52	**0.003** †	0.10	0.46	0.65	**0.002** †	−0.001	0.82	−0.10	0.21	0.004	0.89
L rostral middle frontal	−0.05	0.62	−0.51	**0.001** †	0.09	0.43	0.32	0.07	−0.01	0.01	−0.10	0.15	−0.03	0.24
R superior temporal	0.05	0.57	−0.49	**0.002** †	0.24	0.05	0.47	0.01	−0.01	0.02	−0.10	0.16	0.03	0.16
L postcentral	−0.04	0.61	−0.47	**<0.0001** †	0.26	0.01	−0.04	0.80	−0.01	0.02	−0.01	0.83	0.000	0.98
L caudal middle frontal	−0.04	0.69	−0.43	**0.003** †	0.18	0.12	0.25	0.15	−0.01	0.02	−0.06	0.39	0.01	0.83
L fusiform	−0.03	0.75	−0.42	**0.014** †	0.16	0.24	0.23	0.26	−0.003	0.54	−0.07	0.41	−0.004	0.87
L supramarginal	−0.09	0.32	−0.42	**0.003** †	0.16	0.13	0.08	0.63	−0.01	0.05	−0.12	0.07	0.02	0.47
R caudal middle frontal	−0.13	0.16	−0.41	**0.006** †	0.25	0.03	−0.02	0.91	−0.01	**0.001** †	0.06	0.40	0.02	0.33
R precentral	0.12	0.14	−0.40	**0.002** †	0.37	**<0.0001** †	−0.03	0.84	−0.01	0.004	−0.06	0.32	0.01	0.59
R superior parietal	0.11	0.17	−0.39	**0.003** †	0.18	0.07	0.16	0.29	−0.01	0.04	−0.12	0.06	0.003	0.86
L superior parietal	−0.00	0.98	−0.35	**0.002** †	0.09	0.32	−0.09	0.48	−0.004	0.10	0.01	0.90	0.002	0.92

R, right hemisphere; L, left hemisphere; Infection = number of culture proven infection; Pain = number of skin-breaking procedures exposure; Ventilation = number of days on mechanical ventilation; Morphine = cumulative daily dose in milligrams adjusted for daily body weight; SNAP-II = score for neonatal acute physiology; Surgery = number of surgeries; GA = gestational age.

B values are unstandardized. Number of days on mechanical ventilation was winsorized (replaced the outlier value with the closest value within the ±3 standard deviation range) [84].

† Bold text represents statistical significance; *p*-value threshold for significance adjusted for multiple comparisons with a false discovery rate (FDR) correction set at 5%.

After adjusting for neonatal clinical factors including neonatal pain-related stress, and correcting for multiple comparisons (FDR 5%), greater morphine exposure was associated with increased cortical thickness in bilateral caudal anterior cingulate (*p* = 0.000 right; *p* = 0.003 left), right posterior cingulate (*p* = 0.002), as well as in the left lingual (*p* = 0.002) and middle temporal regions (*p* = 0.002) (Table 6). In our NICU, very preterm infants only received morphine if they underwent mechanical ventilation (n = 25). To confirm these GENLIN results, we reran the analysis on the 25 infants exposed to mechanical ventilation. Morphine remained significant only in the right caudal anterior and posterior cingulate cortices in the subset of ventilated very preterm children.

**Table 6:** GENLIN results for the 5/66 cortical regions significantly associated with neonatal cumulative morphine exposure (adjusted for clinical confounders).

	Infection		Pain		Ventilation		Morphine		SNAP-II		Surgery		GA	
Brain Regions	B	*p*-value	B	*p*-value	B	*p*-value	B	*p*-value	B	*p-*value	B	*p*-value	B	*p-*value
R caudal anteriorcingulate	0.23	0.07	−0.03	0.89	0.09	0.57	0.95	**<0.0001** †	0.004	0.45	−0.27	0.004	0.08	0.01
L caudal anteriorcingulate	−0.02	0.89	−0.25	0.34	0.27	0.18	0.89	**0.003** †	−0.002	0.75	−0.09	0.45	0.11	0.003
L middle temporal	−0.09	0.44	−0.18	0.34	−0.09	0.51	0.68	**0.002** †	−0.01	0.01	−0.11	0.22	0.04	0.10
L lingual	0.01	0.90	−0.52	**0.003** †	0.10	0.46	0.65	**0.002** †	−0.001	0.82	−0.10	0.21	0.004	0.89
R posterior cingulate	0.08	0.40	−0.26	0.10	0.30	0.02	0.58	**0.002** †	−0.002	0.55	−0.25	0.001	0.05	0.04

R, right hemisphere; L, left hemisphere; Infection = number of culture proven infection; Pain = number of skin-breaking procedures exposure; Ventilation = number of days on mechanical ventilation; Morphine = cumulative daily dose in milligrams adjusted for daily body weight; SNAP-II = score for neonatal acute physiology; Surgery = number of surgeries; GA = gestational age.

B values are unstandardized. Number of days on mechanical ventilation was winsorized (replaced the outlier value with the closest value within the ±3 standard deviation range) [84].

† Bold text represents statistical significance; *p*-value threshold for significance adjusted for multiple comparisons with a false discovery rate (FDR) correction set at 5%.

## Discussion

Our primary finding was that greater neonatal pain-related stress (adjusted for multiple clinical factors related to preterm birth and NICU care) was associated with lower cortical thickness at school age in children born very preterm. Specifically, in a conservative two-step approach, first using constrained principal component analysis followed by generalized linear modeling to control for clinical confounders related to prematurity, we found that pain-related stress was the strongest predictor of the variation in overall cortical thickness. Precisely, after applying a 5% false discovery rate to control for overall Type-I error, and adjusting for neonatal clinical factors, greater exposure to neonatal skin-breaking procedures was the predominant consistent neonatal factor related to thinner cortex in 21/66 areas, specifically in frontal, parietal, and temporal regions, independent of the neonatal confounding factors.

Consistent with our present study, we recently found in a different cohort of preterm infants, after similarly controlling for multiple confounding neonatal clinical factors, that greater exposure to neonatal pain-related stress was associated with altered brain microstructural development (subcortical gray matter and white matter) from early in life to term equivalent age [8]. It is known that during the last trimester of gestation and early postnatally, major axonal development takes place in the cerebrum, and damage to white matter tracts (i.e. decreased myelination) and subcortical structures leads to detrimental effects on neuronal migration and cortical development [30], [54]. Thus, in two independent cohorts, our work provides converging evidence that higher exposure to neonatal pain-related stress may contribute to altered brain development early in life and later at school-age in children born very preterm.

Prematurity has been associated with short and long-term abnormalities in cortical thickness compared to healthy term born controls [7], [13]–[18]. Lax *et al.* [7] showed lower overall mean cortical thickness in 25 very preterm 9 year-old children compared to 32 full-term controls, with significant cortical thinning in bilateral precuneus and right supplemental motor cortex, anterior insula, superior temporal, and postcentral regions. Compared to 22 control children born at full-term, Zubiaurre-Elorza *et al.* [14] found reduced cortical thickness in left middle and superior frontal regions, supramarginal, and post central gyri in 14 preterm 9 year-old children with no evidence of PVL. In the present study, we found lower cortical thickness was related to greater neonatal pain exposure in these same regions among children born very preterm. Thus it appears we have identified a potential factor that may contribute to the etiology of reduced cortical thickness in very preterm children. Moreover, our current findings of an association between neonatal pain-related stress (adjusted for clinical confounders) and cortical thickness are consistent with our previous reports on short-term [8] and long-term [55] brain development. Importantly we excluded children with severe brain injury (i.e. PVL and/or IVH grade 3–4) in the newborn period, and/or major neurodevelopmental, motor or sensory impairments, therefore our findings are not explained by severe brain injury. Testosterone levels play a role in cortical thickness in adolescence [53]. In the present study, in 7 year olds born very preterm we did not find differences in cortical thickness between boys and girls in preliminary analyses. Gender was not included as a predictor in our statistical modeling due to limited sample size, and remains for future studies in preterm pre-adolescents.

Thinner cortex in specific regions in our sample of very preterm children may be related to delayed neuronal or glial maturation or cell death, or both. Normal cortical maturation shows an increase in cortical thickness during childhood (up to age 11 years) in most of the lateral frontal, temporal, parietal and occipital regions, a reduction during adolescence, followed by stabilization in adulthood [56]. Hence it is possible that normally occurring cortical growth is delayed in children born very preterm. Recent findings in a premature fetal sheep model showed the importance of cell dysmaturation rather than cell loss (especially neuronal) during cortical growth and microstructure development [54]. The potential to normalize the maturation of these cells remains unknown. Although mechanisms underlying cortical thinning in preterm school-age children remain unclear, findings in rat pup pain models [57]–[59] stress the long-term damaging effects of repetitive neonatal pain exposure on cortical and subcortical neuronal development. However, since we did not compare cortical thickness of very preterm children with a term born control group, it is not possible at this time to conclude that relative reduction in cortical thickness among the very preterm children reflects delayed or accelerated cortical development.

Moreover, volumetric MRI and diffusion tensor imaging (DTI) analyses have demonstrated decreased neuronal volumes (e.g. in cerebellum, thalamus, cerebral cortex) and possible axonal disturbances in very low birth-weight neonates from term-equivalent age to later in adulthood [30]. Sensorimotor, premotor, temporal and parieto-occipital regions are generally reported as the cortical regions showing the most significant volumetric reductions in preterm born school-age children and adolescents [6], [9], [60]–[62]. Smith and colleagues [2] found that greater exposure to stressful procedures (e.g. heel lance/venipuncture, intubation/extubation, diaper change) in the NICU was associated with reduced brain size in the frontal and parietal regions in preterm neonates assessed at term equivalent age. In addition, in that study, functional connectivity MRI and DTI measures showed that alteration in brain microstructure and functional connectivity within the temporal lobes were related to greater stress exposure. Thus, signifying of the importance of early adverse stressful and painful experience converge.

In response to heel lance, Slater *et al.* [63] showed that greater neuronal activity was evident in premature neonates compared to age-matched term-born counterparts. This increased neuronal excitation could be detrimental to the preterm neonate’s immature and rapidly developing neural circuitry by altering apoptosis (programmed cell death) and neuronal survival [64]. However, our understanding of how neonatal pain and stress exposure impacts neurodevelopment in these immature infants is still emerging and basic animal research is crucial to determine mechanisms.

To our knowledge, only a few studies in the neonatal rodent model have specifically examined pain and altered brain development. Early inflammatory pain in neonatal rodent pups induced increased cortical and subcortical neuronal activation [59], increased hippocampal gene expression [58], and widespread cell death [57], [59], thus modifying both the structure and function of the developing brain [57]. Persistent inflammatory pain (formalin injections) or repetitive pain (saline injections) may induce major neuronal apoptosis and altered expression of neurodevelopmentally important proteins in the rat pup brain during the first week of life [57]. The most degenerative cells were found in the lamina II of both frontal and parietal cortex. However, there were differences in long-term effects on the adult brain depending on the timing and type of neonatal pain [57]. Given that the majority of the neonatal pain-related stress procedures occur within the first weeks of the NICU stay, especially in those who undergo the most procedures [8], addressing the management of procedural pain-related stress to protect the developing brain in very preterm infants is pressing. Moreover, opioids seem to have differing effects in the presence or absence of pain. In the Duhrsen et al. study [57] and in previous findings in neonatal rat models [23], [65], pre-emptive morphine only protected the neonatal pup against long-term brain and/or behavioral changes when inflammatory pain was present. In the present study, neonatal cumulative morphine exposure was not related to variation in cortical thickness in 20 of the 21 brain regions significantly adversely associated with pain-related stress exposure. In contrast, greater neonatal morphine was associated with thicker cortex in the cingulate cortices, regions known to be involved in mediating opioid analgesia [66]–[69], as well as in processing emotional aspects of pain [66], [70]–[72]. However, this relationship remained significant only in 2/66 regions when examined in the subset of children who had been exposed to mechanical ventilation and to morphine as a neonate. Thus this finding may reflect Type-I error.

Routine treatment of pain with morphine is no longer advocated in ventilated preterm infants in the NICU [73], since in the short-term continuous morphine infusions lead to longer duration of mechanical ventilation and longer time to reach full enteral feeding [74], and concerns remain about long-term effects of analgesia and sedation on the developing brain [73]–[78]. Our present findings of an association between higher exposure to neonatal morphine and greater cortical thickness in the cingulate regions should be considered in the context of the lack of significant effect in the rest of the cortex. It remains for future research to verify whether these findings imply delayed or accelerated cortical development in very preterm infants at school age. Randomized controlled clinical trials of short [74], [75] and long-term effects [76], [79], [80] of pre-emptive morphine infusion for mechanically ventilated neonates have shown no beneficial effects in the short-term outcomes [74], [75]. Longer-term cognitive/motor and behavioral outcomes in relation to morphine exposure have reported mixed results [76], [79], with the most recent findings suggesting a possible neuroprotective effect of pre-emptive morphine infusion on some aspects of parent report of executive functions at 8–9 years [80]. However, in that study, morphine was not found to be beneficial on teacher reports or on individual testing of child executive functions, thus the results of that study [80] remain tentative.

Importantly, in the present study, severity of illness on day 1 (SNAP-II) also contributed to lower cortical thickness at school age. We have recently found, in a separate cohort, that illness severity of the very preterm neonate during the first 24 hours of life together with pain-related stress exposure contributed to slower microstructural development of the corticospinal tract up to term equivalent age [81]. Together, these findings suggest the importance of protecting the developing brain during the first days of life.

During their NICU care, preterm neonates are exposed to multiple factors that may alter the developing brain, and teasing out specific pain-related effects is challenging. Prenatal and post-natal clinical factors and treatments might interact or may lead to similar end points, which makes them difficult to isolate [82]. Nonetheless, one strength of the present study is extensive control for multiple confounding neonatal clinical factors in our statistical modeling. Unique to the present study of this cohort is the use of constrained principal component analysis as a first step which allowed an unbiased examination of the contribution of 7 pre-selected neonatal clinical factors on cortical thickness variation in 66 brain regions of interest. Our group has shown that neonatal pain-related stress remained a robust predictor of white and gray matter deficits in the short-term [8]. On brain imaging at school age in the present study, the number of skin-breaking procedures during NICU care stood out as the strongest predictor of cortical thickness. Interestingly, we found that greater number of skin-breaks was highly related to bilateral thinner cortex of the postcentral region where the somatosensory cortices lie. Given the known plasticity of the developing pain system [64], associated sensitization, and findings of altered fMRI response to pain at school age [83] it appears highly possible that repeated early noxious nociceptive stimulation may cause rewiring of the somatosensory neural circuitry leading to wider spread detrimental effects on cortical development. Further studies are needed to examine cortical thickness alterations and neonatal pain-related stress exposure in relation to pain threshold and specific domains of cognitive and motor functioning at school age.

This study has limitations in that there could be other confounding factors associated with early pain-related stress exposure and/or prematurity, and a relatively limited sample size. Moreover, the lack of a control group of term born children is a limitation of this study. Advances in prenatal ultrasound have permitted reliable dating of GA. Currently GA is viewed as the single best index of physiological maturity in the preterm newborn, and is thus more widely used as a predictor of long-term outcomes than BW [2], [7]. In contrast, BW reflects a combination of immaturity and potential growth retardation. Examining pain-related stress in infants born small for gestational age (i.e. lower BW than expected relative to GA) in relation to long-term brain development remains to be examined in future studies. Important strengths of our study are that we controlled for seven neonatal factors that capture the most salient neonatal clinical features, the strict exclusion criteria, and a false discovery rate of 5%, thus a conservative approach that reduced the chance of finding significant relationships between neonatal pain-related stress and cortical thickness at school age.

## Conclusion

In very preterm children without severe brain injury in the newborn period, and free from major sensory, motor or neurodevelopmental impairments, we showed that greater exposure to neonatal pain-related stress was associated with thinner cortex in multiple regions at school age, independent of other neonatal risk factors. Important to further advancing our understanding of the relationship between pain-related stress and alterations in cortical development will be the examination of corticospinal tracts, white matter and sub-cortical gray matter structures in school-age children born very preterm. Finally, it remains for future studies to evaluate to what extent these pain-related stress brain alterations are related to cognitive, motor, and behavioral outcomes in these children.
